# Comparison of the short-chain fatty acids in normal rat faeces after the treatment of *Euphorbia kansui*, a traditional Chinese medicine for edoema

**DOI:** 10.1080/13880209.2020.1755318

**Published:** 2020-04-30

**Authors:** Dongjing Jiang, Sijia Guo, An Kang, Yonghui Ju, Jingxian Li, Sheng Yu, Beihua Bao, Yudan Cao, Yuping Tang, Li Zhang, Weifeng Yao

**Affiliations:** a School of Pharmacy, Suzhou Vocational Health College, Suzhou, China; b Jiangsu Key Laboratory for High Technology Research of TCM Formulae, National and Local Collaborative Engineering Center of Chinese Medicinal Resources Industrialization and Formulae Innovative Medicine and Jiangsu Collaborative Innovation Center of Chinese Medicinal Resources Industrialization, Nanjing University of Chinese Medicine, Nanjing, China; c Key Laboratory of Shaanxi Administration of Traditional Chinese Medicine for TCM Compatibility, Shaanxi University of Chinese Medicine, Xi’an, China

**Keywords:** Euphorbiaceae, stir-fried with vinegar, toxicity, biological sample, volatile fatty acids

## Abstract

**Context:**

As a toxic traditional Chinese medicine for edoema, *Euphorbia kansui* S.L. Liou ex S.B. Ho (Euphorbiaceae) (EK) stir-fried with vinegar for detoxification was associated with alterations of gut microbiota. However, the evidence of correlation between short-chain fatty acids (SCFAs) and toxicity of EK has not been confirmed.

**Objective:**

In order to study the biological basis of detoxification of EK stir-fried with vinegar (VEK), a rapid, sensitive and validated GC-MS method was developed to determine SCFAs in normal rat faeces after given EK and VEK.

**Materials and methods:**

Sprague Dawley rats were orally administered 0.5% CMC-Na (control group), EK (EK-treated group) and VEK powder (VEK-treated group) at 680 mg/kg for six consecutive days (eight rats each group). Fresh faeces samples were promptly collected, derivatized and then analyzed by GC-MS.

**Results:**

The ranges of LOD and LOQ were within 0.13–1.79 and 0.45–5.95 μg/mL, respectively. The RSD values of intra-day and inter-day precisions were less than 15%. Four SCFAs were generally stable under four storage conditions. The extraction recoveries were ranged from 53.5% to 97.3% with RSD values lower than 15%. The concentrations of four SCFAs in EK and VEK were decreased significantly compared with those not administered (EK-treated, *p* < 0.01; VEK-treated, *p* < 0.05 and *p* < 0.01). After being stir-fried with vinegar, the concentrations were all increased (*p* < 0.05 and *p* < 0.01).

**Discussion and conclusions:**

The negative correlation between SCFAs and toxicity of EK may provide evidence for biological mechanism and toxic Chinese medicine.

## Introduction

As a kind of traditional Chinese medicine, the dried root of *Euphorbia kansui* S.L. Liou ex S.B. Ho (Euphorbiaceae) (EK) has been widely applied for treating edoema, ascites and pleural fluid effusion in all ages (Shen et al. 2017; Yu et al. 2018). However, inflammation (Shu et al. 2010), liver injury (Tang et al. 2012), intestinal irritation (Gao et al. 2015) and other toxic effects (Tang et al. 2014; Cary et al. 2016) seriously restrict its clinical application. Stir-frying with vinegar as a detoxification method recorded in Chinese Pharmacopoeia is regularly used to reduce the toxicity of EK (Commission 2015). The literature and previous research by our group confirmed that stir-fried with vinegar could reduce the significant gastrointestinal toxicity of EK, while retaining the purgative effect (Cheng et al. 2015; Lou et al. 2018). The toxicity and effect sites of EK have close relation with intestinal tract (Jiang et al. 2018), but results of existing research could not answer the biological basis of EK stir-fried with vinegar (VEK) for detoxification.

Short-chain fatty acids (SCFAs) deficiency may be associated with intestinal toxicity (Stumpff 2018). Known as volatile fatty acids, the carbon atoms of SCFAs are less than seven. SCFAs mainly include acetic acid, propionic acid, butyric acid, and valeric acid produced by microbial fermentation (such as lactic acid bacteria and bifidobacteria) of dietary fibre, resistant starch, oligosaccharides and other unabsorbed dietary carbohydrates (Zhao et al. 2017). SCFAs play important roles in intestinal oxidation energy supplying (Valdes et al. 2018), antitumor (Louis et al. 2014), antipathogenic microorganisms (Kelly et al. 2015) and anti-inflammatory (Huang, Zhou, et al. 2017). Clinical studies suggest that SCFAs can better maintain intestinal morphology and promote sodium absorption. Among them, propionate and butyrate could inhibit proliferation and induce apoptosis of colon cancer cells (Tang et al. 2011). Acetate and butyrate inhibit glomerular mesangial cells proliferation induced by high glucose and reverse the production of reactive oxygen species and malondialdehyde (Huang, Guo, et al. 2017). Acetate, propionate and butyrate transported by MCT-1 suppress TNF-α-induced inflammatory signalling in intestinal cells (Hung and Suzuki 2018). As the direct products of microbial fermentation (Xu et al. 2017), the convenient and rapid determination of SCFAs in faeces is indispensable. Gas chromatography-mass spectrometry (GC-MS) is the most commonly employed methods for measuring SCFAs which is reliable and inexpensive. However, existing methods are usually not considering the influence of the biological sample matrix (Han et al. 2018; He et al. 2018), lead to low repeatability and stability in practical applications.

In this study, a method for quantitative analysis of SCFAs in rat faecal samples was established (mainly accredited to Zheng et al. 2013) and the linear relationship, intra-day and inter-day precisions, stability and extraction recovery were all investigated under the influence of biological sample matrix. Also, this method was well applied in comparing of SCFAs in normal rats’ faeces after treatment of EK and VEK.

## Materials and methods

### Testing samples

The roots of EK (voucher specimen No. NJUTCM-20151115) were collected from Red River valley of Bao ji (Shaanxi, China) in November 2015 and deposited in the Herbarium of Nanjing University of Chinese Medicine (Nanjing, China). The herb was identified by Professor Yuping Tang (Shaanxi University of Chinese Medicine, Xianyang, China). VEK was obtained by immersing 100 g of cleaned EK in 30 g vinegar and then stir-fry at 260 °C until slight scorched spots appeared, 9 min approximately. The dried EK and VEK were ground into powder and sieved through a 100 mesh stainless steel sieve before use.

### Animal and biological sample collection

SPF male Sprague Dawley rats weighing (230 ± 20) g were purchased from Zhejiang Experimental Animal Center (license number: SCXK 2014-0001, China), and housed at a certified animal experimental laboratory. All of the studies on animals were conducted in accordance with the guidelines of the Animal Ethics Committee of Nanjing University of Chinese Medicine.

After adaptation feeding for 1 week, the rats were randomly assigned to three groups (eight rats per group): control group, EK-treated group, VEK-treated group. The control group was given 0.05% CMC-Na (1 mL/100 g), and the EK-treated group and VEK- treated group were given 680 mg/kg (8-fold concentration amount at clinical dosage) of EK and VEK powder, respectively. They were orally treated once per day, for 6 days. Fresh faeces samples (2–4 tablets) were collected on 6th day after administration, then frozen immediately in liquid nitrogen and stored at –80 °C. Each portion of frozen 100 mg faecal samples was put in a 2 mL tube (Corning, NY, USA) and thawed gradually on ice before use.

### Chemicals and reagents

Acetic acid (Lot No. C0027517, ≥99.7%) and propionic acid (Lot No. A15J7L9136, ≥99.5%) were purchased from Dr. Ehrenstorfer (Augsburg, Germany). Butyric acid (Lot No. 755WP0-JS, ≥99.8%) and valeric acid (Lot No. FGN01-JLBK, ≥99.0%) were purchased from TCI Development Co., Ltd. (Shanghai, China). Hexanoic-6,6,6-d3 acid (Lot No. ZZS17102803, 99.0%) was purchased from Shanghai Zzbio Co., Ltd. (Shanghai, China). Sodium hydroxide (Lot No. 160928, ACS grade) was purchased from Xilong Scientific Co., Ltd (Beijing, China). 1-Propanol (Lot No. LP700111, HPLC grade) and pyridine (Lot No. LB30R04, HPLC grade) were purchased from J&K Scientific Ltd. (Beijing, China). Propyl chloroformate (Lot No. 11502046, HPLC grade) and hexane (Lot No. E1725082, HPLC grade) were purchased from Shanghai Aladdin Bio-Chem Technology Co., Ltd. (Shanghai, China).

### Preparation of derivatization solution of faecal sample

#### Extraction of faecal sample

The extraction procedures of faecal samples were performed at 4 °C to minimize the loss of volatile SCFAs. 0.005 mol/L aqueous NaOH (1 mL) containing internal standard (IS-hexanoic-6,6,6-d3 acid) was added to each faecal sample (100 mg). A 500 μL aliquot of supernatant was transferred into a 10 mL glass centrifuge tube after vibrated for 10 min and centrifuged for 20 min (12,000 *g*, 4 °C). Water (300 μL) was then added to get an 800 μL mixture.

#### Derivation of faecal extract

An aliquot of 500 μL 1-propanol/pyridine mixture solvent (v/v = 3:2) and 100 μL of propyl chloroformate were subsequently added and was vortexed briefly. After derivatization, the derivatives samples were extracted by a two-step extraction with hexane. Hexane (300 μL) was added to the sample, vortexed for 1 min, and then centrifuged for 5 min (2000 *g*, 4 °C). The upper layer (200 μL) was transferred to a 1.5 mL centrifuge tube. Another 200 μL of hexane was added to the sample and the extraction repeated and combined with the previous upper layer. The resultant mixture was centrifuged for 5 min (2000 *g*, 4 °C) and 200 μL of the supernatant was taken to GC-MS analysis.

### Preparation of standard solutions and is solution

Acetic acid, propionic acid, butyric acid, valeric acid and IS were accurately weighed in each 10 mL volumetric flask. Four standard stock solutions and IS stock solution were prepared individually at concentrations of 18.23, 8.849, 16.99, 2.807 and 1.258 mg/mL, respectively. Aqueous NaOH solution (0.005 mol/L) was added to dilute IS stock solution until concentration at 5.032 μg/mL. All solutions were stored at 4 °C before use.

### GC–MS analysis

The GC-MS analysis was performed on an Agilent 7890B gas chromatograph coupled with an Agilent 7000C mass spectrometric detector (Agilent Technologies, Santa Clara, CA). A HP-1 ms (5% phenyl-95% methylpolysiloxane) capillary column (30 m × 0.25 mm i.d., 0.25 μm film thickness, Agilent Technologies) with carrier gas (helium) at a flow rate of 1 mL/min was utilized for SCFAs separation. Sample (1 μL) was injected into the system with a split ratio of 10:1. The oven programme was set at 50 °C for 2 min, raised to 70 °C at a rate of 10 °C/min, to 85 °C at a rate of 3 °C/min, to 110 °C at a rate of 5 °C/min, to final temperature of 290 °C at a rate of 30 °C/min and maintained for 8 min.

The temperatures of the front inlet, transfer line and electron impact (EI) ion source were set at 260, 290 and 230 °C, respectively. The electron energy was 70 eV, and the mass spectral data was collected in a full scan mode (*m*/*z* 30–600). The detection parameters of each component were shown in Table 1.

**Table 1.** Detecting parameter of each component.

Component	Molecular weight	Retention time (min)
Acetic acid	60	2.69
Propionic acid	74	3.90
Butyric acid	88	5.47
Valeric acid	102	7.96
IS	116	11.00

### Method validation

The developed method was validated for linearity, sensitivity, precision, stability and extraction recovery according to the guidelines of Chinese Pharmacopoeia (2015 Edition).

#### Linearity and sensitivity

Two tablets of each faecal sample (*n* = 8) collected from control group were mixed as biological sample matrix. A series of standard mixture solutions (I–VI) were diluted with ultrapure water to different concentrations for plotting standard curves (Table 2). Biological sample matrix (100 mg) was accurately weighed to a 2 mL centrifuge tube, 100 μL of each standard mixture solution (I–VI) was precisely added; the 1 mL of 0.005 mol/L aqueous NaOH was added. A 550 μL aliquot of supernatant with an IS concentration of 5.030 μg/mL was transferred into a 10 mL glass centrifuge tube. Water (250 μL) was then added to get an 800 μL mixture. The mixture was derivatized refer to above method ‘Fecal extract derivation’, then injected and analysed. The calibration equation took weighted partial least squares method (weight, 1:C^2^). The *Y* was standard concentration, and the *X* was：*X* = (*A*_matrix + standard_ – *A*_matrix_)/*A*_IS_. Faecal matrix containing standard mixture solution I, III and VI were set as high, middle and low quality control samples, respectively. The limits of detection (LOD) and quantification (LOQ) were calculated based on the peak-to-noise ratio of 3:1 and 10:1, respectively. For each target constituent, the LOD and LOQ were determined by serial dilution of the standard solution under the described GC-MS conditions.

**Table 2.** Concentration values of level I–VI (μg/mL).

Compounds	I	II	III	IV	V	VI
Acetic acid	5469	2735	341.9	171.0	85.50	42.75
Propionic acid	1770	885.0	110.6	55.30	27.65	13.83
Butyric acid	3398	1699	212.4	106.2	53.10	26.55
Valeric acid	280.7	140.4	17.55	8.775	4.388	2.194

#### Intra-day and inter-day precisions

The instrument precision was confirmed by the measurement of the intra-day variances and inter-day variances. The intra-day precision was assessed by five replications of the high- middle- and low-quality control samples within 1 day. The inter-day precision was assessed by three replications of the high-, middle- and low-quality control samples for 3 days.

#### Stability

This experiment investigated the stability of quality control samples under four storage conditions, i.e., short-term stability, freeze-thaw stability, prepared samples stability and long-term stability. Test samples of short-term stability were stored at room temperature for 0, 24 and 48 h, respectively. Test samples of freeze-thaw stability were stored at room temperature 12 h and at –20 °C 12 h three times. Test samples of prepared sample stability were stored at sampler for 2 h. Test samples of long-term stability were stored at –70 °C for 15 days. Each test sample was separated into four aliquots and stored in the corresponding condition.

#### Extraction recovery

Peak area ratio of each compound from five aliquots of faecal matrixes were calculated and recorded as A. Peak area ratio of each compound from five aliquots of quality control samples were calculated and recorded as B. The extraction recovery of each compound was counted as B/A.

### Data processing

Agilent MassHunter Qualitative Analysis software (B.07.00) was used to process the GC-MS data for peak picking, standard curve construction, and SCFAs quantification. The concentration of SCFAs in faeces were calculated using the calibration equations constructed from the GC-MS data. Two-tailed Student’s *t*-test was used for statistical comparisons. Statistical analyses were performed using the Statistical Package for Social Science programme (SPSS 24.0, Chicago, USA). *p* Values < 0.05 were considered statistically significant.

## Results

### Procedure optimization

Pre-experiment was carried out to investigate the contents of acetic, propionic, isopropyl, butyric, 2-methylbutyric, valeric and heptanoic acids in faeces derived from normal rats gavaged with EK. It was found that isopropyl, 2-methylbutyric and heptanoic acids were not detected in the faeces. For this, acetic acid, propionic acid, butyric acid and valeric acid were selected as study subjects in this experiment. There were many methods for detecting SCFAs in biological samples such as serum (Zhao et al. 2007), urine (Jain et al. 2015), saliva, platelets (Bao and Mao 1991), intestinal contents and faecal culture fluid (Lu et al. 2014). These existing methods directly selected aqueous solution to investigate the extraction of SCFAs. The effect of matrix such as faeces on the content of SCFAs were not fully considered in the actual process. Based on the full consideration of biological sample matrix (faeces), propyl chloroformate was taken as an esterified derivatizing reagent. Reaction time of this method was short and volatilization of SCFAs could be effectively avoided in a low temperature environment. The derivatization process was carried out in the aqueous phase, facilitating the processing of biological samples. Moreover, the derivatives were easy to obtain and the testing samples did not need to be re-separated. The operation process became simpler and easier. At the same time, derivatized reagents were relatively inexpensive, which could effectively reduce the costs of this experiment.

### Method validation

The calibration equations, correlation coefficients, linearity range, LOD and LOQ for the listed four compounds were shown in Table 3. It indicated that they had a good linearity and the ranges of LOD and LOQ were within 0.13–1.79 and 0.45–5.95 μg/mL, separately.

**Table 3.** Calibration equations, *r* values, LOQ and LOD for four compounds.

Compounds	Calibration equation	Correlation coefficient (*r*)	Linear range (μg/mL)	LOD (μg/mL)	LOQ (μg/mL)
Acetic acid	*Y* = 155.27*X* – 173.60	0.9973	42.75–5469	0.46	1.55
Propionic acid	*Y* = 59.261*X* – 37.084	0.9927	13.84–1770	1.23	4.11
Butyric acid	*Y* = 37.493*X* – 19.318	0.9940	26.55–3398	1.79	5.95
Valeric acid	*Y* = 21.385*X* – 10.210	0.9902	2.194–280.7	0.13	0.45

The results of precision were presented in Table 4. The RSD values of intra-day and inter-day precisions were less than 15% for each analyte which indicated that the precision of this method was well acceptable. These compounds were generally stable under four storage conditions (Table 5). The extraction recoveries of four compounds ranged from 53.5% to 97.3% with RSD values lower than 15%, demonstrating that the method was accurate and feasible, the extraction recovery data were shown in Table 6.

**Table 4.** Inter-day and intra-day precisions.

Compounds	Concentration (μg/mL)	Intra-day precision RSD (%) (*n* = 5)	Inter-day precision RSD (%) (*n* = 9)
Acetic acid	5469	3.4	7.2
	341.9	8.8	7.8
	42.75	14.3	13.9
Propionic acid	1770	9.8	10.2
	110.6	12.1	9.8
	13.83	9.1	11.0
Butyric acid	3398	7.9	8.1
	212.4	9.9	8.2
	26.55	11.2	10.8
Valeric acid	280.7	11.5	9.7
	17.55	9.6	9.9
	2.194	13.2	14.8

**Table 5.** Stability investigation (*n* = 3).

Compounds	Concentration (μg/mL)	Short-term stability RSD (%)			Freeze-thaw stability RSD (%)	Prepared samples stability RSD (%)	Long-term stability RSD（%）
		0 h	24 h	48 h			
Acetic acid	5469	3.4	5.0	6.0	7.6	2.3	12.1
	341.9	7.5	12.9	4.1	8.6	7.6	11.5
	42.75	12.4	3.49	8.2	9.4	3.5	14.2
Propionic acid	1770	6.8	14.4	4.0	5.3	11.5	13.8
	110.6	12.6	6.1	11.7	4.9	10.1	7.2
	13.83	10.1	7.8	10.4	9.4	7.8	7.9
Butyric acid	3398	10.8	11.4	2.8	2.3	7.0	10.6
	212.4	3.8	7.6	8.1	8.6	7.3	5.7
	26.55	12.0	12.8	8.0	6.1	12.8	10.0
Valeric acid	280.7	7.8	7.7	9.9	2.1	9.0	5.3
	17.55	11.7	3.0	13.3	3.2	8.4	7.0
	2.194	11.3	13.5	9.2	11.3	13.5	3.3

**Table 6.** Extraction recovery of short-chain fatty acids and IS (*n* = 5).

Compounds	Concentration (μg/mL)	Extraction recovery (%)	Extraction recovery RSD (%)
Acetic acid	5469	94.5	6.43
	341.9	74.0	6.98
	42.75	97.3	6.85
Propionic acid	1770	89.3	5.66
	110.6	71.7	10.2
	13.83	79.8	13.7
Butyric acid	3398	95.3	9.51
	212.4	77.5	9.64
	26.55	53.5	13.2
Valeric acid	280.7	91.0	12.0
	17.55	90.0	13.8
	2.194	92.9	6.15
IS	5.032	92.0	11.7

### Quantification and comparison of four SCFAs in EK and VEK

**Figure 1.**
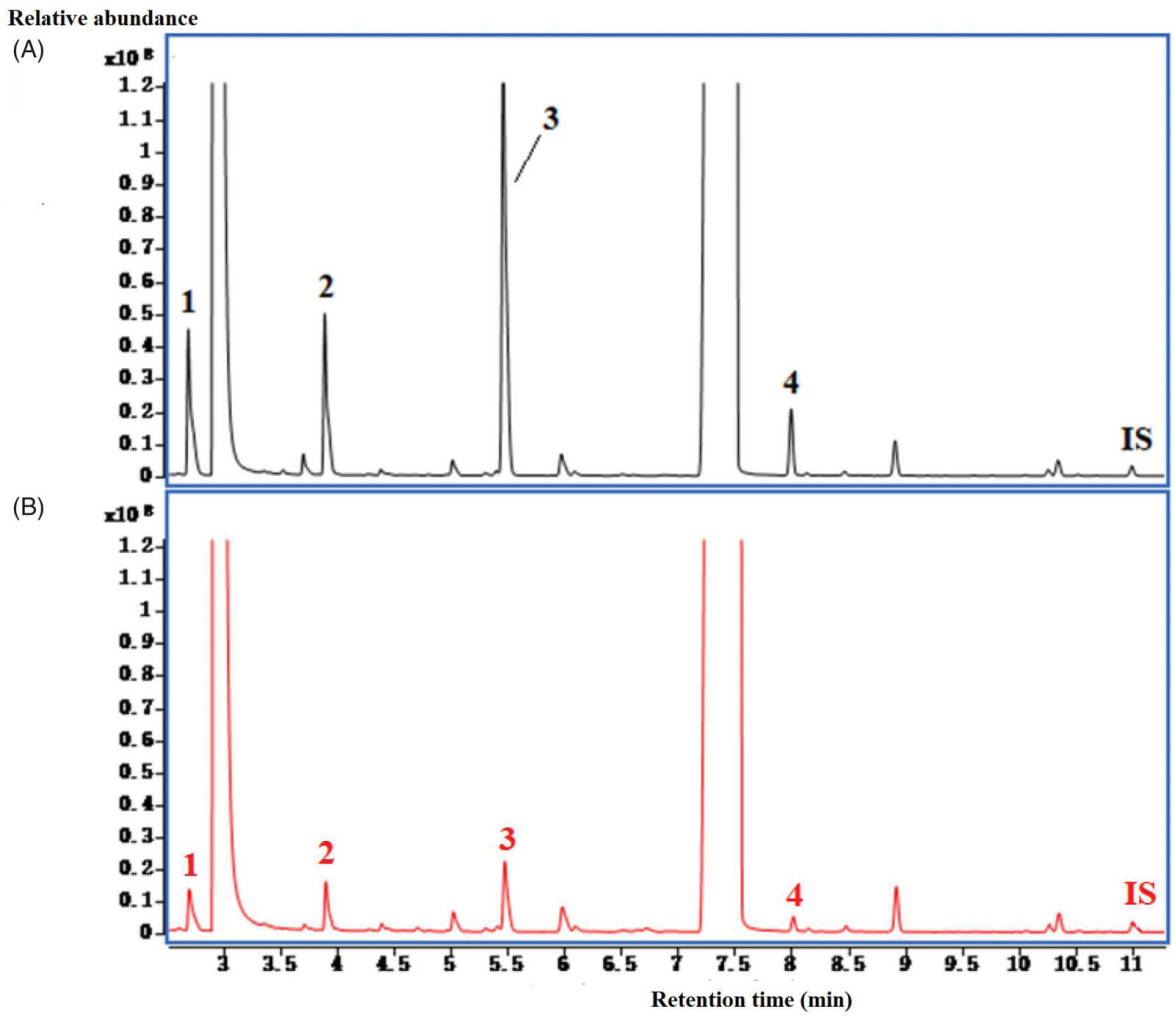
Typical GC-MS chromatography of faeces spiked with standard mixture solutions (A) and faecal sample (B) Peak identification: 1, acetic acid; 2, propionic acid; 3, butyric acid; 4, valeric acid; IS.

The GC-MS method was successfully applied for the quantification of four SCFAs in EK and VEK. The typical chromatogram of faeces spiked with standard mixture solutions (A) and faecal sample (B) were shown in the Figure 1. The concentrations of four SCFAs in EK and VEK were listed in Tables 7 and 8. Compared with control group, the concentrations of acetic acid, propionic acid, butyric acid and valeric acid were significantly decreased in EK-treated group and VEK-treated group (EK-treated, *p* < 0.01; VEK-treated, *p* < 0.05 and *p* < 0.01). Compared with EK-treated group, the concentrations of these four SCFAs were significantly increased to different degree of VEK-treated group (*p* < 0.05, *p* < 0.01). Previous research of our group confirmed that EK stir-fried with vinegar could significantly reduce the contents of toxic components originated from ingenane-type diterpenoids and jatrophane-type triterpenoids (Zhang et al. 2017, 2018). Liver function injury, inflammatory damage and other toxic side effects could also be weakened when EK stir-fried with vinegar (Lou et al. 2018). SCFAs could regulate liver injury (Hu et al. 2018) and inhibit inflammatory (Ashton et al. 2017). The SCFAs had an anti-inflammatory effect by inhibiting the recruitment and proinflammatory activity of neutrophils, macrophages, dendritic cells and effector T cells and by increasing the number and activity of regulatory T cells (Gonçalves et al. 2018). In this study, low concentrations of SCFAs in EK and high concentrations of SCFAs in VEK also successfully proved the above viewpoint. The contents of these four SCFAs showed a demotion in the group with high toxicity and a promotion in the group with low toxicity.

**Table 7.** Quantification for SCFAs by GC-MS analysis of faecal samples in rats (μg/mL).

Compounds	Control	EK-treated	VEK-treated
Acetic acid	3168 ± 407.0	1026 ± 80.28**	2215 ± 196.6**^△△^
Propionic acid	751.5 ± 150.3	315.8 ± 49.29**	574.0 ± 109.3*^△^
Butyric acid	1602 ± 571.0	542.3 ± 219.6**	1147 ± 226.5^△^
Valeric acid	84.94 ± 23.07	27.62 ± 8.408**	60.84 ± 19.61**^△^

Data are shown as the means ± SD (*n* = 8 rats per group). *p < 0.05, **p < 0.01 when compared with the control group, ^△^*p* < 0.05, ^△△^*p* < 0.01 when compared with the EK-treated group.

**Table 8.** Determination of four kinds of SCFAs in faecal samples.

Group	Number	Acetic acid			Propionic acid			Butyric acid			Valeric acid		
		Peak area ratio	Concentration (μg/mL)	Mean (μg/mL)	Peak area ratio	Concentration (μg/mL)	Mean (μg/mL)	Peak area ratio	Concentration (μg/mL)	Mean (μg/mL)	Peak area ratio	Concentration (μg/mL)	Mean (μg/mL)
Control	1	22.59	3334	3168	9.090	501.6	751.5	45.50	1687	1062	4.427	84.45	84.94
	2	18.67	2725		15.94	907.7		29.63	1091		3.783	70.69	
	3	18.35	2676		12.15	683.1		36.18	1337		4.038	76.15	
	4	18.26	2662		14.85	842.8		29.51	1087		3.103	56.14	
	5	24.55	3638		14.31	810.9		59.16	2199		4.141	78.35	
	6	23.4	3460		16.54	942.8		41.57	1539		6.422	127.1	
	7	23.21	3430		11.26	630.4		32.49	1199		3.995	75.23	
	8	23.14	3419		12.32	692.8		71.93	2677		5.687	111.4	
EK-treated	1	7.375	971.5	1026	6.262	334.0	315.8	15.37	557.0	542.3	2.014	32.85	27.62
	2	7.348	967.3		5.520	290.0		9.854	350.1		1.598	23.97	
	3	7.282	957.0		5.500	288.9		20.42	746.4		2.240	37.70	
	4	7.278	956.5		6.657	357.4		7.511	262.3		1.454	20.88	
	5	8.474	1142		6.747	362.7		13.62	491.2		1.198	15.41	
	6	8.468	1141		4.847	250.1		25.71	944.6		2.256	38.03	
	7	8.004	1069		5.053	262.4		15.68	568.6		1.474	21.32	
	8	7.573	1002		7.047	380.5		11.67	418.4		1.917	30.78	
VEK-treated	1	15.35	2210	2215	9.447	522.8	574.0	33.44	1234	1147	2.778	49.20	60.84
	2	14.87	2135		8.830	486.2		33.83	1249		3.899	73.17	
	3	14.00	2000		10.86	606.5		30.26	1115		2.207	36.99	
	4	13.55	1931		8.621	473.8		21.06	770.2		2.543	44.17	
	5	15.39	2215		12.87	725.8		32.78	1210		2.509	43.43	
	6	17.41	2529		11.40	638.6		40.44	1497		4.632	88.84	
	7	16.46	2383		12.46	701.4		32.91	1215		4.222	80.08	
	8	16.04	2317		8.002	437.1		24.19	887.8		3.791	70.85	

## Discussion and conclusions

In this study, acetic acid, propionic acid, butyric acid and valeric acid of faeces were quantitatively tested by GC-MS method. This method has a limit of quantitation of 0.45–5.95 μg/mL and proved to be accurate, simple, stable and high in extraction recovery, which could be well applied in biological samples. Then, the concentrations of these four SCFAs were explored in the normal rat faeces after treatment of *E. kansui* before and after stir-fried with vinegar. The concentrations of these four SCFAs on EK treated rats were decreased significantly compared with those not administered and were all increased after stir-fried with vinegar. Combined with the changes in the toxicity, it revealed the content of SCFAs and toxicity of EK maybe negatively correlated.
